# Study on Masking the Bitterness of Chinese Medicine Decoction-Mate

**DOI:** 10.1155/2022/3701288

**Published:** 2022-09-09

**Authors:** Fuguo Hou, Yan Miao, Xiangxiang Wu, Xinjing Gui, Yanli Wang, Haiyang Li, Junhan Shi, Lu Zhang, Jing Yao, Xuelin Li, Ruixin Liu

**Affiliations:** ^1^School of Pharmacy, Henan University of Chinese Medicine, Zhengzhou 450046, China; ^2^Department of Pharmacy, The First Affiliated Hospital of Henan University of Chinese Medicine, Zhengzhou 450000, China; ^3^The Level Three Laboratory of Chinese Traditional Medical Preparation of State Administration of Traditional Chinese Medicine, Zhengzhou 450000, China; ^4^Henan Province Engineering Research Center for Clinical Application, Evaluation and Transformation of Traditional Chinese Medicine, Zhengzhou 450000, China; ^5^Co-Construction Collaborative Innovation Center for Chinese Medicine and Respiratory Diseases By Henan & Education Ministry of China, Henan University of Chinese Medicine, Zhengzhou 450000, China; ^6^Henan Key Laboratory for Clinical Pharmacy of Traditional Chinese Medicine, Zhengzhou 450000, China

## Abstract

**Background:**

Traditional Chinese medicine decoction (TCMD) is an oral liquid made by decocting crude medicinal compounds with water. It has complex compositions and diverse odor and taste, most of which have an unacceptable level of bitterness which seriously affects patients' medication compliance. To solve this problem, a variety of taste-masking pathways and different types of taste-masking excipients were combined, using the application of coffee-mate to mask the bitterness of coffee as an existing example. Three composite taste-masking adjuvants were developed to improve the taste of TCMD, referred to as the Chinese Medicine Decoction-Mate (CMD-M). However, whether CMD-M has a good taste-masking effect and whether it affects the chemical compositions and pharmacological effects of the medicine remain unclear.

**Method:**

The commonly used pediatric medicine Qingre Huazhi Decoction (QRHZD) and the personalized decoctions used in clinical practices were used as the masking research carriers. The taste-masking effect of CMD-M on QRHZD was evaluated by both healthy volunteers and an electronic tongue, and the personalized decoctions were evaluated by clinical subjects. The changes of chemical components of QRHZD before and after taste-masking were evaluated by HPLC. The changes in anti-inflammatory effects were evaluated by establishing mice as an acute inflammatory model.

**Results:**

The taste-masking effect evaluation results showed that the bitterness of QRHZD was significantly reduced after adding CMD-M. There was no significant difference in the relative peak areas change rate and total peak areas ratio of common peaks of QRHZD before and after taste-masking (*P* > 0.05), shown by HPLC analysis. The inhibitory rates of QRHZD on ear swelling in mice before and after taste-masking also showed no significant difference (*P* > 0.05).

**Conclusions:**

CMD-M can effectively mask the bitterness of decoctions while bringing no significant difference overall in chemical compositions and pharmacological effects before and after QRHZD masking.

## 1. Introduction

Traditional Chinese medicine decoction (TCMD) is a compound oral liquid preparation made by adding crude medicines into water and removing the scum after boiling. The dosage form has been widely used in Asian countries, such as China, Japan, the Republic of Korea, and the Democratic People's Republic of Korea. However, this dosage form has the characteristics of large doses per patient, complex compositions per dose, and diverse odor and taste. Most traditional Chinese medicine (TCM) has an associated bitterness. TCMs such as *Citrullus Colocynthis* [1], *Coptis chinensis* [2], and *Sophora flavescens* [3] contain active pharmaceutical ingredients (APIs) as well as alkaloids [4], glycosides, and flavonoids, which have an especially unacceptable bitterness [5], while TCMs such as honey [6], jujube [7], and hawthorn [8] accounted for a smaller proportion of TCMs with an acceptable taste. These characteristics lead to the poor taste of the final preparation, which seriously restricts the application of TCMD. Therefore, it is of great significance to study the masking of bitterness to improve patients' medicine compliance [9].

Coffee is a drink with a refreshing effect. However, it has a bitterness that is unacceptable to many people, which affects the degree of its popularity. In order to improve its taste, coffee-mate was created. This not only reserved the aroma but also reduced the bitter taste of coffee, and is thoroughly enjoyed by many people. TCMD has some similarities with coffee in terms of its complex compositions and bad taste. Therefore, the idea of developing Chinese Medicine Decoction-Mate (CMD-M) of three flavors (sweet orange, chocolate, and coffee) to cover up the bitterness of TCMD was devised.

**Figure 1:**
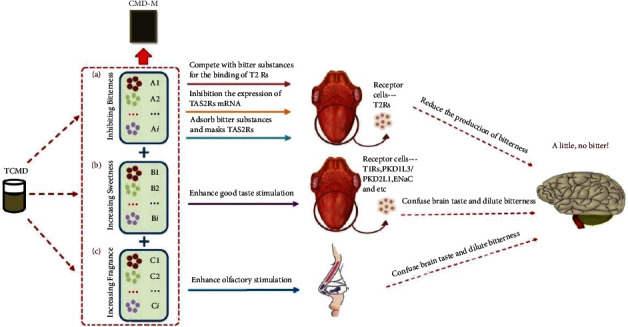
Schematic diagram of TCMD three taste-masking methods.

In the present field of medicine [10], the main methods for inhibiting bitterness include adding flavoring agents [11] or bitterness inhibitors [12], forming inclusion complexes [13], granulating [14], preparing microcapsules or microspheres [15], forming solid dispersions [16], and preparing precursor substances [17]. Taste-masking methods have been frequently used for a long time in the field of chemical medicine. In recent years, the methods described above to inhibit the bitterness pathway has also achieved initial success when transplanted to the taste-masking of solid preparations of traditional Chinese medicine. However, due to the complex compositions of TCMDs, the research on inhibiting TCMD's bitterness has been unusual until now. Based on the formation mechanisms of bitterness and the inhibition of bitterness mechanisms, a multiprinciple and multidimensional taste-masking strategy was proposed. As shown in Figure 1, there are three main taste-masking methods: inhibiting bitterness, increasing sweetness, and increasing fragrance. Inhibiting bitterness means directly or indirectly blocking the production of bad taste through a variety of ways, increasing sweetness is to confuse or mask bad taste stimulation through good taste stimulation, and increasing fragrance to mask bad taste stimulation through using good olfactory stimulation.

Qingre Huazhi Decoction (QRHZD), a commonly used prescription in pediatrics, has anti-inflammatory and antipyretic pharmacological effects. In this study, QRHZD was used as the research carrier. The traditional human taste panel method (THTPM) [18] and electronic tongue method [19] were used to evaluate the taste-masking effect and clinical application effect of three CMD-Ms. In addition, chemical composition changes and pharmacological effects of QRHZD before and after taste-masking were investigated by HPLC and animal experiments in order to provide a theoretical basis for the clinical application of CMD-M.

## 2. Materials and Methods

### 2.1. Drugs and Reagents

Granular extracts of 11 kinds of crude drugs, including *Radix Bupleuri*, *Radix Scutellariae*, *Radix Puerariae*, Gypsum, *Rhizoma Anemarrhenae*, *Cortex Magnoliae Officinalis*, *Areca catechu*, Raw coix seed, *Notopterygium*, *Aurantii Fructus Immaturus*, and licorice were purchased from Jiangyin Tianjiang Pharmaceutical Co. Ltd. (Jiangyin, China). Dexamethasone acetate tablets were purchased from Zhejiang Xianju Pharmaceutical Co. Ltd. (Taizhou, China). Physiological saline was purchased from Shijiazhuang Siyao Co. Ltd. (Shijiazhung, China). Toluene (GB/T64941996) was purchased from Tianjin Yongda Chemical Reagent Co. Ltd. (Tianjin, China). Baicalin reference substance (batch number:110715-201318) and Liquiritin reference substance (batch number:111610-201106) were purchased from the China Institute for the Control of Pharmaceutical and Biological Products (Beijing, China). Acetonitrile and phosphoric acid were chromatographically pure and purchased from Sigma (American Sigma Company). Methanol and ethanol were analytically pure chemicals. Ultrapure water was purchased from Millipore (Millipore Q, 18.2 MΩ·cm).

### 2.2. Preparing Chinese Medicine Decoction-Mate

Sweet orange flavor CMD-M was composed of sucrose, sucralose, *β*-CD, sweet orange fruit powder, citric acid, malic acid, and orange essence extract. Among these, sucrose, citric acid, and malic acid were crushed, sucralose was mixed evenly with citric acid, malic acid, and sweet orange fruit powder by the equal increment method, and then mixed evenly with sucrose powder and *β*-CD. The mixture was dried at 55–65°C and cooled to room temperature (18–25°C). Orange essence extract was added and mixed evenly.

Chocolate flavor CMD-M was composed of sucrose, sucralose, *β*-CD, barley malt extract, milk powder, cocoa powder, maltodextrin, vanilla essence extract, and salt.

Coffee flavor CMD-M was composed of sucrose, sucralose, *β*-CD, coffee, and nondairy creamer.

All three CMD-Ms have authorized Chinese patents, and the patent numbers are CN103800914B, CN103800908B, and CN103766557B, respectively.

### 2.3. Evaluation of the Taste-Masking Effect of CMD-Ms

#### 2.3.1. Taste Evaluation Based on Healthy Volunteers

*(1) Volunteer Screening*. This study was approved by the Ethics Committee of the First Affiliated Hospital (ECFAH) of the Henan University of Chinese Medicine with approval number 2017HL-066-01. The research team conducted a strict screening of recruited volunteers, including the sensitivity to bitterness, among other traits. Inclusion criteria were as follows: (1) As bitterness perception decreases with age, adults around 20 years old were selected [20]; (2) There could be no history of severe allergies, genetic disease, cholecystitis, drug or alcohol abuse, and no recent history of the disease. Exclusion criteria included the following: (1) People who had consumed alcohol or smoked in the last 2 days; (2) People who were too nervous; (3) Volunteers were screened on their reactions to a lemon-yellow aqueous solution that contained no bitterness: volunteers who reported a bitter taste were excluded; (4) People who had eaten within 3 hours before tasting the solution, especially food that had a strong flavor. In this study, 20 healthy volunteers (9 males and 11 females) were selected, and all volunteers gave informed consent and signed an informed consent form.

Standardized training for volunteers' taste evaluation: referring to the methods found in the literature [21], the bitterness was divided into 5 levels. Each level was given a certain range of bitterness values, and different concentrations of berberine hydrochloride solution were prepared as the bitterness reference solution (Table 1). Lemon yellow coloring was added to make these solutions similar in color. 20 mL of berberine hydrochloride reference solution with different mass concentrations were obtained and put into mouth-tasting cups at 37°C. The volunteers put this solution in their mouth for 15 s and performed the mouth washing action in the oral cavity to make the bitterness perception areas at the root and side of the tongue perceive the bitterness of the sample. Then the volunteers compared the bitterness classification and specific bitterness value of the reference solution according to the description in Table 1. After the reference solution was spat out, volunteers cleaned their mouths with distilled water at least 3 times until bitterness was no longer felt in the oral cavity. After 15 minutes, another concentration of the reference solution was measured, and the standardized sensory memory of bitterness was completed.

The QRHZD original drug liquid was prepared according to the composition ratio of QRHZD (*Radix Bupleuri* 36 g, *Radix Scutellariae* 20 g, *Radix Puerariae* 20 g, Gypsum 60 g, *Rhizoma Anemarrhenae* 20 g, *Cortex Magnoliae Officinalis* 12 g, *Areca catechu* 20 g, raw coix seed 60 g, *Notopterygium* 12 g, *Aurantii Fructus Immaturus* 12 g, and licorice 18 g, with all measurements corresponding to the quantity of Chinese herbal pieces). The granular extracts of each of the natural medicines were weighed. Each prescription was added to 180 mL water and put into a suitable container, then placed on the induction cooker to heat and dissolve the drugs. The decoction was cooled to room temperature for further taste-masking preparation.

The QRHZD taste-masking drug liquid was prepared by taking an appropriate amount of the QRHZD original drug liquid and adding the different flavors CMD-Ms. QRHZD was marked as S, and three flavors of CMD-Ms were marked as coffee, sweet orange, and chocolate. Then the taste-masking drug liquid after adding CMD-M was marked as *S* + coffee (10.0 g/bag/180 mL), *S* +  sweet orange (9.0 g/bag/180 mL), and *S* + chocolate (7.0 g/bag/180 mL).

Then, the 20 healthy volunteers evaluated the bitterness of the above-mentioned QRHZD original drug liquid and taste-masking drug liquids based on their taste and the bitterness reference solution. The specific tasting operation requirements were the same as the above-mentioned standardized training requirements. Finally, the volunteers recorded the bitterness grade and specific bitterness value of the sample after tasting. The evaluation results were analyzed by the integrated score evaluation method.

Since there may be individual differences between different volunteers in the taste experiment, there will be individual abnormal values in the test data. Therefore, referring to the literature [22], the Grubbs test method was used to perform loop inspection and eliminate the outliers in the data. In this experiment, the detection level was 0.1 and the elimination level was 0.05.

### 2.4. Taste Evaluation Based on Clinical Subjects

This study was approved by the Pediatric Center of Henan University of Chinese Medicine (approval number: 2017HL-066-01). The research team recruited subjects in the second and fifth wards of the Pediatric Inpatient Department of the First Affiliated Hospital of the Henan University of Chinese Medicine on a voluntary basis by visiting, surveying, and consulting the medical staff and parents in the pediatric ward regarding the opinions of the subjects. According to the characteristics of the trial population, 96 clinical subjects (59 males and 37 females) were finally selected as subjects. The subjects selected different flavors of CMD-Ms according to their own wishes, and signed an informed consent form before the test.

Due to the different conditions of the subjects and their medications, it is recommended to add one packet of CMD-Ms to every 200 mL of TCMD. The evaluation method was to inform subjects or their family members of different levels of bitterness and bitterness descriptions, then have the subjects themselves evaluate the bitterness of their taken TCMDs and describe the taste-masking effect of CMD-Ms. The taste-masking effect could be compared with the taste of the original TCMD for evaluation, and the predesigned “clinical trial evaluation form” (Table 2) was filled in. The complete information collection form for subjects is shown in Attached list-Table 1.

### 2.5. Taste Evaluation Based on Electronic Tongue

To prepare the positive electrode cleaning solution, 7.46 g of potassium chloride was accurately weighed and dissolved with 500 mL of distilled water. Then 300 mL of absolute ethanol solution and 0.56 g of potassium hydroxide were added while stirring. After dissolving, the solution was transferred to a 1000 mL volumetric flask and adjusted to the final volume.

The negative electrode cleaning solution was prepared by mixing 300 mL of absolute ethanol with 500 mL of distilled water by shaking, then adding 8.3 mL of concentrated hydrochloric acid and mixing followed by transferring to a 1000 mL volumetric flask and adjusting to the final volume.

The reference solution was prepared by weighing 2.24 g of potassium chloride and 0.045 g of tartaric acid, then dissolving them with 500 mL of distilled water. The solution was transferred to a 1000 mL volumetric flask and adjusted to the final volume.

The preparation of the sample to be tested was the same as the preparation method of the taste evaluation sample described above.

The TS-5000Z taste sensor (smart sensor technology company) was used for measurement. Firstly, the sensor was cleaned in the cleaning solution for 90 s, then rinsed in the reference solution twice for 120 s each time. The sensor was then returned to zero in the balance position for 30 s. After reaching the balance condition, the test was started. The test time was 30 s before the first taste value was output. Then, the sensor was briefly cleaned in the two groups of reference solutions for 3 s and inserted into the new reference solution to test the aftertaste for 30 s. This test was performed 4 times. The first time sample was removed, and the average value of the last 3 times was taken as the test results. The liquids of each wash, balance, and test the aftertaste were distributed in different sample cups.

*(1) Electronic Tongue Sensor Selection*. The sensors of the TS-5000Z electronic tongue used in this experiment were C00, AE1, CA0, CT0, and AAE, respectively. Among them, C00, AE1, and AEE sensors were shown to have two kinds of bitterness information, called the first taste and the aftertaste, while CA0 and CT0 sensors only output the first taste value (Table 3).

### 2.6. Evaluation of the Influence of CMD-Ms on Chemical Compositions by HPLC Characteristic Chromatograms

#### 2.6.1. Chromatographic Conditions

HPLC (Waters E2695, Waters, USA) and SB-Aq columns (Agilent ZORBAX, 4.6 × 250 mm, 5 *μ*m) were used for analysis. The mobile phase consisted of acetonitrile (A) and 0.1 mol·L^−1^ phosphoric acid aqueous solution (B), and the elution gradient was 0–6 min, 8%–12% A; 6–45 min, 12%–25% min A; 45–60 min, 25%–50% A; 60–64 min, 50%–90% A. The column temperature was maintained at 25°C. The detection wavelength was 280 nm. The mobile phase flow rate and injection volume were 1.0 mL·min^−1^ and 5 *μ*L, respectively.

#### 2.6.2. Sample Preparation

*(1) The Preparation of Reference Solution*. The baicalin and liquiritin reference substances were weighed and diluted with methanol to prepare a mixed reference solution containing 199 *μ*g baicalin and 202 *μ*g glycyrrhizin per milliliter and then filtered through a 0.45 *μ*m filter membrane.

*(2) The Preparation of QRHZD Test Solution*. The granular extracts of natural medicines were weighed according to the composition ratio of QRHZD and dissolved in 240 mL of hot water. The solution was adjusted to 250 mL after it cooled down. Then, 60 mL of the solution was weighed and put into a 250 mL volumetric flask. 180 mL of ethanol was added to the flask while shaking. The sample was sonicated for 20 min. After cooling, the volume was adjusted with ethanol, and the sample was filtered through the 0.45 *μ*m filter membrane to obtain the QRHZD test solution. Five samples were labeled as S1, S2, S3, S4, and S5, respectively.

The preparation of the QRHZD taste-masking solution was the same as described above, but with minor additions: the QRHZD should be heated to about 60°C in a water bath first, and then 4.2 g of sweet orange flavor CMD-M should be added to the sample. After shaking well, the steps described above should be carried out. Finally, the QRHZD taste-masking solution with sweet orange flavor CMD-M was prepared. The five samples were labeled as S1-S5 + sweet orange. The same method was used to prepare 5 portions of QRHZD taste-masking solution with 5.0 g coffee flavor CMD-M and 5 portions of QRHZD taste-masking solution with 7.0 g chocolate flavor CMD-M.

The preparation of the CMD-M test solution: 4.2 g sweet orange flavor, 5.0 g coffee flavor, and 7.0 g chocolate flavor CMD-M were weighed out, then the same method was used to prepare the QRHZD test solution was applied.

#### 2.6.3. Collection of Characteristic Chromatographic

10 *μ*L of the reference solution and 5 *μ*L each of the test solution and the taste-masking solution were taken, then the samples were injected. All components were collected within 65 min.

### 2.7. Evaluation of the Influence of CMD-Ms on Pharmacological Effects by the Mice Acute Inflammatory Model

With the approval of ECFAH of the Henan University of Chinese Medicine, the research team purchased 60 Kunming mice from the Experimental Animal Center of Zhengzhou University with weights between 18 and 22 g. The mice were kept in plastic cages at room temperature (22 ± 1)°C, light and dark cycle of artificial light for 12 h, and relative humidity of 60 ± 5%. This study was conducted in the Central Laboratory of the First Affiliated Hospital of the Henan University of Chinese Medicine.

The granular extracts of natural medicines were weighed according to the composition ratio of QRHZD and then made into a solution, which was divided into four portions. Sweet orange flavor CMD-M (10.0 g/bag/200 mL), coffee flavor CMD-M (10.0 g/bag/200 mL), and chocolate flavor CMD-M (14.0 g/2 bag/200 mL) were added to the above three portions, respectively. 50 mg of dexamethasone acetate and physiological saline were made into a mixed solution with a mass fraction of 0.0375% and stored until ready for use.

60 Kunming mice were randomly divided into physiological saline blank group, dexamethasone acetate control group, QRHZD group (*S*), QRHZD + sweet orange flavor CMD-M group (*S* + Orange), QRHZD + chocolate flavor CMD-M group (*S* + Chocolate), and QRHZD + coffee flavor CMD-M group (*S* + Coffee). Each group contained 10 mice. The auricle swelling method was used to model inflammation in the mice. The blank group was given physiological saline by gavage. The control group was given 0.75 mg·kg^−1^ mixed solution, which is 15 times the amount of human clinical dosage, by gavage. The other four groups were given corresponding QRHZD mixtures by gavage. Each group was treated once a day for 5 consecutive days. One hour after the last did, 0.02 mL of xylene was applied to the front and back of the left ear of the mice to cause inflammation, and the mice were sacrificed 30 minutes after the inflammation initiation. A 7 mm diameter hole punch (YLS-25A) was used to punch the earpieces at the same part of the mice's left and right ears, and the pieces were weighed with an electronic balance (CF225D). Then the left and right ears were weighed, and the degree of swelling was calculated. The following equations were used to calculate ear measurements:

(1) $$\begin{array}{c} \text{swelling degree}=\text{left ear weight}-\text{right ear weight},\\ \text{swelling rate}\left(\%\right)=\left(\frac{\text{left ear weight}-\text{right ear weight}}{\text{right ear weight}}\right)\cdot 100\%. \end{array}$$

### 2.8. Statistical Analysis

The similarity evaluation system for the chromatographic fingerprint of TCM (Version 2012 A) was used to process the chromatographic data to obtain the characteristic chromatographic. The remaining data were analyzed by SPSS 22.0 statistical analysis software.

## 3. Results

### 3.1. Evaluation Results of the Taste-Masking Effect of CMD-Ms

#### 3.1.1. Taste Evaluation Results Based on Healthy Volunteers

**Figure 2:**
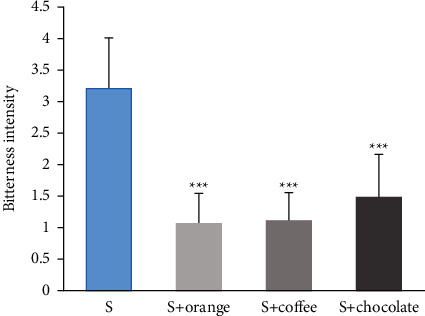
Comparison of bitterness values based on “ranking method + scoring method”(*s*, *n* = 4). Note: ^*∗∗∗*^represents *S* + orange, *S* + coffee and *S* + chocolate respectively compared with *S* (*P* < 0.001). The average of bitterness values of sample *S* + orange, *S* + coffee, *S* + chocolate and S (*X*‾ ± SD) were 1.06 ± 0.49, 1.11 ± 0.45, 1.49 ± 0.68, 3.20 ± 0.81, respectively.

Results were evaluated using the ranking and scoring method (Figure 2). After adding three flavors of CMD-Ms for taste-masking, the bitterness value of QRHZD was significantly different from that of the taste-masked QRHZD when compared using the paired *t*-test (*P* < 0.001). From the bitterness value reduction after taste-masking, the ranked results of three CMD-Ms taste-masking effects were: sweet orange flavor > coffee flavor > chocolate flavor.

#### 3.1.2. Taste Evaluation Results Based on Clinical Subjects

**Figure 3:**
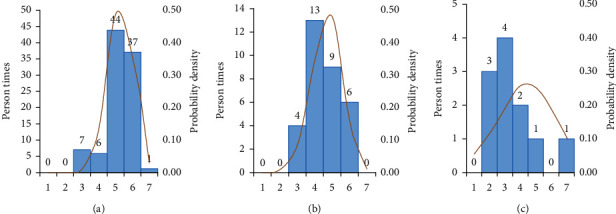
(a–c) The taste-masking effect after adding sweet orange, chocolate, and coffee flavor CMD-M to the TCMD of the subjects in the treatment period.

95 responses were received from the subjects who used the sweet orange flavor CMD-M, 32 responses from the subjects who used chocolate flavor CMD-M, and 11 responses from the subjects who used the coffee flavor CMD-M. Based on the collected responses, the ranked results of three CMD-Ms taste-masking effects were orange flavor > chocolate flavor > coffee flavor (Figure 3). The sweet orange flavor CMD-M was the most popular and the taste-masking scores were mostly concentrated on “5” and “6” (with scores from 1–7. The higher the score, the more popular it was).

#### 3.1.3. Taste Evaluation Results Based on Electronic Tongue

**Figure 4:**
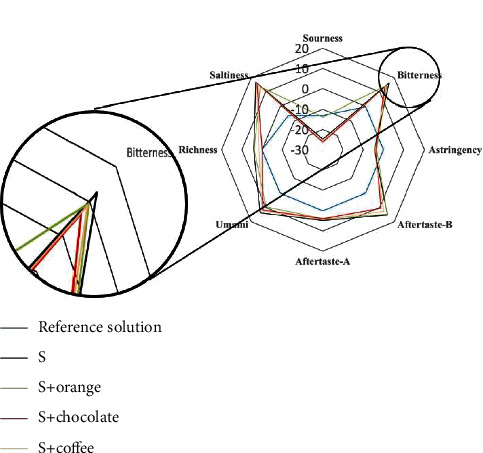
Taste information radar images of different taste-masking samples.

The bitterness response value of QRHZD after adding three flavors of CMD-Ms was lower than that before taste-masking (Figure 4). Among them, the bitterness response value decreased the most after adding chocolate flavor CMD-M, from 16.31 to 13.37, which is an 18.03% decrease. The bitterness response values of adding sweet orange flavor and coffee flavor CMD-M were 14.80 and 14.53, respectively. These results indicate that the three CMD-Ms have a certain taste-masking effect on the bitterness in QRHZD. In addition, the sweet orange flavor CMD-M had a stronger sour taste, so the sourness response value of QRHZD increased significantly after taste-masking, while the sourness response values were slightly reduced in the other two flavors of CMD-Ms. The response value of astringency had also decreased overall, which was conducive to the improvement of the QRHZD taste to a certain extent.

### 3.2. Evaluation Results of the Influence of CMD-Ms on Chemical Compositions by HPLC Characteristic Chromatograms

#### 3.2.1. Methodological Verification

The results of the precision, reproducibility, and stability experiments showed that the relative retention times RSD of the main common peaks was below 0.2%, and the relative peak areas RSD was below 5%, indicating that the precision of the instrument and the reproducibility and stability of the method was sound. The 40.4 *μ*g·ml^−1^, 101.0 *μ*g·ml^−1^, 202.0 *μ*g·ml ^−1^, 242.4 *μ*g·ml^−1^, and 303.0 *μ*g·ml^−1^ of mixed reference solution were injected for the determination of the regression equation. Linear regression was performed on the injection volume X (*μ*g·ml^−1^) with the peak area Y of liquiritin, and the regression equation was obtained: *y* = 2 × 10^6^ X–178677, *R*^2^ = 0.9998. The results showed that the injection volume of liquiritin had a good linear relationship within 40.4∼303.0 *μ*g·ml^−1^.

#### 3.2.2. The Establishment of the Characteristic Chromatographic

**Figure 5:**
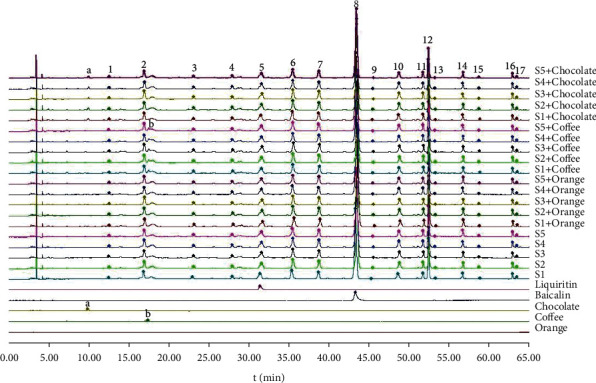
The changes of characteristic chromatogram of QRHZD before and after taste-masking.

The system automatically matched 17 common chromatographic peaks with better resolution and larger peak areas among samples (Figure 5). The figure showed that QRHZD had 17 common peaks before taste-masking and still had 17 common peaks after taste-masking. The coffee flavor CMD-M had a peak at around 17 minutes, and the chocolate flavor CMD-M had a peak at around 10 minutes. The sweet orange flavor CMD-M did not have a detectable peak in this wavelength range.

Peak 8 was taken with better resolution as the reference peak. The retention times ratio and peak areas ratio of each chromatographic peak to the reference peak in the same chromatographic peak were calculated, and the relative retention times and relative peak areas were obtained. For the convenience of statistical analysis, S1, *S*1 + Orange, *S*1 + Coffee, *S*1 + Chocolate, ..., *S*5 + Chocolate were marked as S1, S2 ... S20, respectively (Attached Tables 2 and 3).

### 3.3. Comparison of Changes in Relative Peak Areas of the Common Peaks before and after Taste-Masking

**Figure 6:**
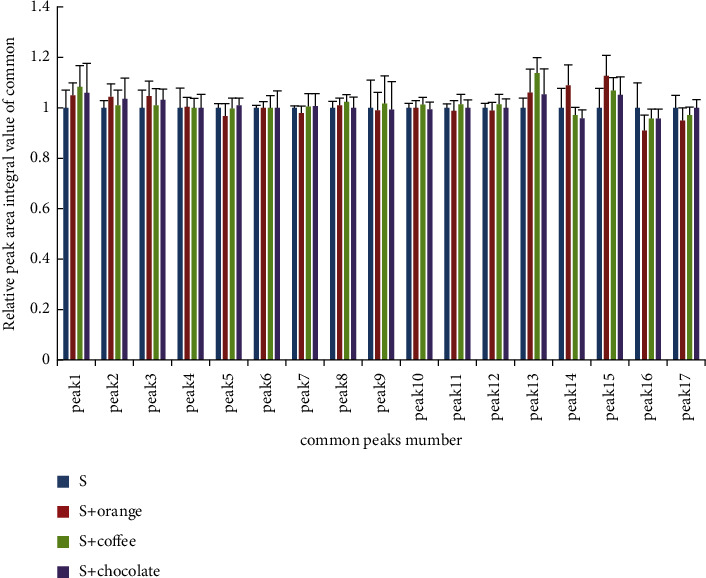
Comparisons of common chromatographic peaks of QRHZD before and after taste-masking (‾*X* ± *S n* = 5).

The relative peak areas of the 17 common peaks of QRHZD test solution as a unit, the average value of the absolute value of relative peak areas change rate of each common peak in the characteristic chromatogram, and the integral value of the relative peak areas was compared. The changes of each peak after taste-masking are shown in Table 4 and Figure 6.

SPSS 22.0 statistical analysis software was used to analyze the relative peak areas change rate. The Shapiro-Wilk test was used to determine whether the taste-masking results conformed to the normal distribution, and the correlation analysis was carried out. These results showed that the absolute value of the relative peak areas change rate of QRHZD after adding three CMD-Ms all conformed to the normal distribution (*P* > 0.05), and the average values of the absolute value of relative peak areas change rate of the QRHZD after taste-masking were 3.56 ± 5.12%, 2.63 ± 3.46%, and 1.86 ± 2.40%, respectively.

Histogram analysis showed that the total areas of the 17 common peaks of QRHZD changed little after taste-masking, but there were individual common peaks that had relatively large changes. There was no significant difference in the peak areas of the 17 common peaks in QRHZD and the peak areas of the common peaks after adding coffee flavor CMD-M by paired *t*-test (*P* > 0.05). The peak areas of the 17 common peaks in QRHZD and the peak areas of the common peaks after adding sweet orange flavor CMD-M were tested by paired *t*-test. The results showed that peaks 14, 15, and 17 had significant differences (*P* < 0.05), and the other peaks had no significant differences (*P* > 0.05). The peak areas of the 17 common peaks in QRHZD and the peak areas of the common peaks after adding chocolate flavor CMD-M were tested by paired *t*-test. Only peak 15 had a significant difference (*P* < 0.05), and the other peaks had no significant differences (*P* > 0.05).

### 3.4. Comparison of Changes in Total Peak Areas of Common Peaks before and after Taste-Masking

The average value of all common peak areas in the chromatogram of QRHZD test solution was set to 1, and the normalization method was used to calculate the ratio of the relative peak areas of QRHZD chemical compositions after the adding three CMD-Ms for taste-masking. The obtained ratio was QRHZD: sweet orange flavor: coffee flavor: chocolate flavor = 1.00 : 0.99 : 0.98 : 1.01, indicating that the total amount of chemical compositions in QRHZD was approximately the same before and after taste-masking.

The quantitative analysis by HPLC characteristic chromatogram showed that there were 51 single peaks (17 × 3) in the samples after adding three CMD-Ms for taste-masking, and 45 of them had the absolute change rate of relative peak areas below 5%, accounting for 88.24%. There were 5 species within 5% to 10%, accounting for 9.80%, of which *S* + sweet orange, *S* + coffee, and *S* + chocolate account for 1.96%, 5.88%, and 1.96%, respectively. Only one was more than 10%, accounting for 1.96% of the change rate. The normalization method was used to compare the total peak areas of common peaks before and after taste-masking. The results showed that the total peak areas of three CMD-Ms had no significant changes before and after taste-masking.

### 3.5. Evaluation Results of the Influence of CMD-Ms on Pharmacological Effects by the Mice Acute Inflammatory Model

**Figure 7:**
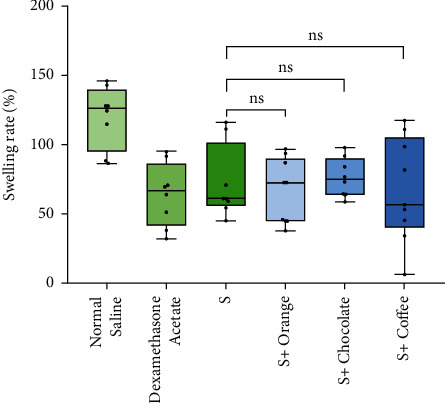
Comparison of anti-inflammatory effects of QRHZD before and after taste-masking. Note: “ns” represents the comparison with the QRHZD group (*P* > 0.05).

The three CMD-M groups all had different degrees of anti-inflammatory effects and had inhibitory effects on xylene-induced ear swelling in mice. Compared with the anti-inflammatory effects of QRHZD, there was no significant difference (*P* > 0.05). The results are shown in Table 5 and Figure 7.

## 4. Discussion

In addition to *Radix Puerariae*, Gypsum, raw coix seed, and ricorice in the prescription of QRHZD, the other seven natural medicines are described as bitter or slightly bitter in the 2020 edition of Chinese Pharmacopoeia [23], which leads to a bitterness of QRHZD that is unacceptable to pediatric patients, thus seriously affecting the compliance of patients taking this decoction. Therefore, in this study, QRHZD was used as the carrier of bitterness to evaluate the taste-masking effect, characteristic chromatography analysis, and pharmacological effects of three flavors of CMD-Ms after optimization.

Currently, the commonly used taste evaluation methods for volunteers in the world include the “single sample comparative evaluation method” [24], “bitterness value grade evaluation method” [25], “integrated score evaluation method(ISEM)” [26], “fuzzy mathematics comprehensive evaluation method” [27], “visual analog scale method” [28], “multifactor survey evaluation method” [29], “measurement matching + amplitude marking evaluation method” [30], and “taste magnetic resonance imaging evaluation method” [31]. Each method has its own unique advantages. Based on the THTPM, this study took the bitterness value of QRHZD before and after taste-masking as an indicator, and the “integrated score evaluation method” was used to evaluate the taste-masking effect of three flavors of CMD-Ms. This method has the advantages of high-ranking accuracy, judgment sensitivity, and fitting degree.

In the evaluation of the taste-masking effect, there were some differences in the taste-masking effect of CMD-M between healthy volunteers and clinical subjects. The reason may be that healthy volunteers were adults aged 22–28 years, and 91% of subjects were children aged 0–13 years. Children are fonder of sweet and sour tastes, so they had a high acceptance of the sweet orange flavor CMD-M with its sweet and sour taste. Another reason may be that the coffee flavor CMD-M contained a small amount of coffee, which has a certain bitterness, and it also contains a small amount of caffeine, which may make it less popular in children, resulting in limited clinical data collection and low-level characterization of the taste-masking effect of the coffee flavor CMD-M. In addition, the clinical subject evaluation experiments used self-tasting controls, which can reduce the influence of subjective factors between individuals compared to single-blind or double-blind and other controlled experiments [32–34]. In the evaluation of the taste-masking effect of the electronic tongue, the chocolate flavor CMD-M had the best taste-masking effect, which was contradictory to the results of healthy volunteers. This may be due to the subjectivity of the THTPM: that is, the evaluation results of different volunteers may have great differences due to their own preferences or prejudices. Therefore, the experimental data of electronic tongue is more objective, which can eliminate the interference of human subjective factors.

In this study, HPLC was used to establish the fingerprint chromatogram of QRHZD before and after taste-masking, and the change rate of relative peak areas and total peak areas of common peaks were used as indicators for quantitative analysis. The results showed that the chemical compositions of QRHZD before and after taste-masking were basically the same, and the three flavors of CMD-Ms had no obvious effect on the chemical compositions. Although the total peak areas of the characteristic chromatogram of QRHZD after adding chocolate flavor CMD-M was basically unchanged compared with that before taste-masking, the peak areas of peak 15 had relatively large changes, which may be caused by the changes of physical properties such as chemical conductivity and pH after adding three CMD-Ms. Its specific chemical structure and whether it has an impact on clinical efficacy still needs to be verified by further experiments.

By consulting relevant information and literature [35], it was found that the main pharmacological effects of QRHZD were anti-inflammatory and antipyretic. The research conducted a preexperimental design for anti-inflammatory effects and antipyretic effects at the early stage. The preexperimental results showed that QRHZD had significant anti-inflammatory effects, but the antipyretic effects were not obvious. Therefore, in the experimental design of pharmacological effects of QRHZD before and after taste-masking, the anti-inflammatory effects were selected to evaluate the changes of pharmacological effects. The results showed that there was no significant difference in anti-inflammatory effects of QRHZD before and after taste-masking (*P* < 0.0), and the three CMD-Ms had no significant influence on anti-inflammatory effects.

The taste-masking methods used in CMD-Ms mainly include inhibiting bitterness, increasing sweetness, and increasing fragrance, in which inhibiting bitterness is defined as directly or indirectly blocking the production of bad taste through a variety of ways. The components of three CMD-Ms all contain *β*-CD, which is a cyclic hollow compound with internal hydrophobicity and external hydrophilicity. Therefore, it can form inclusion complexes with hydrophobic bitter groups, thereby reducing the number of drugs in contact with taste buds in the process of ingestion and, to a certain extent, reducing bitterness. Increasing sweetness was used to confuse or mask bad taste stimulation through good taste stimulation. The compositions of three CMD-Ms all contained sucrose and sucralose, which can increase the sweet sensation and confuse the taste sensation of the brain, thereby achieving the effect of masking the bitterness. Increasing fragrance was also used to mask bad taste stimulation through good olfactory stimulation. Three flavors of CMD-Ms were added with corresponding flavors, respectively, all of which achieved a good fragrance enhancement effect. In this study, the CMD-M with sweet orange flavor was more popular with child patients due to its sour and sweet taste. The CMD-M was based on the combined application of three taste-masking methods of inhibiting bitterness, increasing sweetness, and increasing fragrance to achieve TCDM taste-masking from many aspects. Compared to the taste-masking agents based on only one method, the taste-masking effect of all three methods will be better. The CMD-Ms developed in this study had three flavors of sweet orange, chocolate, and coffee. Patients can choose their favorite flavor according to their own conditions, which can greatly improve the acceptance of TCDMs in patients.

## 5. Conclusions

Through this study, it was found that the bitterness of QRHZD was significantly reduced after adding CMD-M for taste-masking and that the chemical compositions and pharmacological effects of QRHZD before and after taste-masking had no significant changes overall, indicating that CMD-M can improve the oral applicability of the decoction without affecting the properties of medicines. The development and application of CMD-Ms are of great significance in solving the problems that have plagued the poor taste of TCMD for thousands of years, improving patients' drug-taking compliance, and increasing the application rate of TCMD.

Developing and innovating safe and effective bitterness inhibitors will solve the problems of bitterness and poor taste of TCMD, but whether the taste-masking will affect the pharmacokinetics of TCMD is not yet known. Whether there are side effects caused by the medicine after adding CMD-Ms or the side effects of the TCMD itself is still yet to be studied, many clinical trials need to be conducted to verify the safety of these mixtures.

## Figures and Tables

**Table 1:** Bitterness grades and mass concentration of corresponding reference solution.

No.	Description of intensity of bitterness	Rank assigned	Corresponding scale	Conc of corresponding reference samples
1	Imperceptible	I	[0.5–1.5)	0 mg·mL^−1^
2	Slight	II	[1.5–2.5)	0.01 mg·mL^−1^
3	Moderate	III	[2.5–3.5)	0.05 mg·mL^−1^
4	High (but still acceptable)	IV	[3.5–4.5)	0.10 mg·mL^−1^
5	Extreme (almost acceptable)	V	[4.5–5.5]	0.50 mg·mL^−1^

**Table 2:** Clinical trial evaluation form.

No.	Description of taste-masking effect	Evaluation (“√”)
1	More pain than not adding.	□ I
2	Similar to not adding, basically did not cover up the bitterness.	□ II
3	Better than not, the bitterness is somewhat reduced.	□ III
4	Better than not, the bitterness has dropped a lot, but it is still a bit bitter.	□ IV
5	Basically not bitter!	□ V
6	Not only does it not bitter, but also sweetness, not bad!	□ VI
7	I like the taste! it can be enjoyed as a drink!	□ VII

**Table 3:** Sensors taste information.

Sensor	Corresponding taste	Taste information	
		First taste	Aftertaste
C00	Acidic bitterness	Bitterness	Aftertaste-B
AE1	Astringency	Astringency	Aftertaste-A
CA0	Sourness	Sourness	—
CT0	Saltiness	Saltiness	—
AAE	Umami	Umami	Richness

**Table 4:** The change rate of the relative peak areas of QRHZD before and after taste-masking.

Peak number	*S*	*S* + orange	Change rate (%)	*S* + coffee	Change rate (%)	*S* + chocolate	Change rate (%)
1	1	1.026	2.592	1.053	5.275	1.039	3.911
2	1	1.041	4.084	0.992	−0.802	1.035	3.506
3	1	0.990	−0.996	0.969	−3.141	0.993	−0.733
4	1	1.040	4.039	1.010	0.996	1.021	2.122
5	1	0.958	−4.162	0.976	−2.388	1.007	0.723
6	1	0.993	−0.683	0.994	−0.649	1.021	2.076
7	1	0.973	−2.729	0.986	−1.409	1.009	0.867
8	1	1.000	0.000	1.000	0.000	1.000	0.000
9	1	0.994	−0.586	1.027	2.733	0.998	−0.200
10	1	0.995	−0.532	0.992	−0.752	0.997	−0.304
11	1	0.985	−1.513	0.990	−0.956	1.005	0.495
12	1	0.983	−1.677	0.991	−0.940	1.004	0.432
13	1	1.018	1.754	1.070	7.016	1.025	2.479
14	1	1.088	8.789	0.958	−4.168	0.969	−3.123
15	1	1.138	13.762	1.062	6.187	1.069	6.895
16	1	0.921	−7.912	0.952	−4.757	0.975	−2.535
17	1	0.954	−4.636	0.975	−2.528	1.012	1.198
The SD values of change rate			5.124		3.457		2.390
The average absolute values of the change rate			3.556		2.629		1.859

**Table 5:** Comparison of anti-inflammatory effects of QRHZD before and after taste-masking.

Group	*n*	Dosage (g/kg)	Swelling degree (mg)	Swelling rate (%)
Normal saline	8	—	12.58 ± 2.30	120.00 ± 22.45
Dexamethasone acetate	8	0.00075	6.11 ± 2.25	64.14 ± 22.83
S	8	14.18	7.46 ± 2.62	72.66 ± 26.56
*S* + orange	9	14.18 + 0.91	7.80 ± 3.18	66.06 ± 23.27^ns^
*S* + chocolate	8	14.18 + 1.27	8.34 ± 1.73	76.47 ± 14.17^ns^
*S* + coffee	9	14.18 + 0.91	7.41 ± 3.94	67.30 ± 37.52^ns^

*Note*. “ns” represents the comparison with the QRHZD group (*P* > 0.05).

## Data Availability

All data, models, and code generated or used during the study are included within the article.

## Abbreviations

TCMD: Traditional Chinese medicine decoction
TCM: Traditional Chinese medicine
CMD-M: Chinese medicine decoction-mate
QRHZD: Qingrehuazhi decoction
THTPM: Traditional human taste panel method
ECFAH: Ethics Committee of the First Affiliated Hospital.

## Supplementary Materials

Supplementary Materials Attached Table 1 is the clinical trial evaluation form, which includes the basic information of clinical subjects such as name, age, gender, and disease, as well as the description of drug bitterness, a brief introduction of filling in the form, and the options for subjects to evaluate the taste-masking effect rating of CMD-M. Attached Table 2 shows the original data of the relative retention time of the common chromatographic peaks in sample solutions before and after QRHZD taste-masking. Attached Table 3 shows the original data of the relative peak areas of the common chromatographic peaks in sample solutions before and after QRHZD taste-masking.
