# Supplementary material for: Study on Masking the Bitterness of Chinese Medicine Decoction-Mate
Source: Evid Based Complement Alternat Med. 2022 Sep 9;2022:3701288. doi: 10.1155/2022/3701288 (PMC9481366; doi:10.1155/2022/3701288)
Supplement: Supplementary Materials — Attached Table 1 is the clinical trial evaluation form, which includes the basic information of clinical subjects such as name, age, gender, and disease, as well as the description of drug bitterness, a brief introduction of filling in the form, and the options for subjects to evaluate the taste-masking effect rating of CMD-M. Attached Table 2 shows the original data of the relative retention time of the common chromatographic peaks in sample solutions before and after QRHZD taste-masking. Attached Table 3 shows the original data of the relative peak areas of the common chromatographic peaks in sample solutions before and after QRHZD taste-masking. [file 3701288.f1.zip › Attached list--Table1.pdf]

Table 1 Clinical trial evaluation form

Clinical Validation Evaluation Form

Test date：　　　　　　　　　　　　　　Subject number：

Name	Gender	Age	Telephone number	Disease name	Chinese Medicine Decoction-Mate	□ Sweet orange-flavor CMD-M<br>□ Chocolate-flavor CMD-M<br>□ Coffee-flavor CMD-M
Drug type	□ traditional decoction<br>□ dispensing granule decoction		composition of the prescription		History of drug allergy	

Number	Taste description	Bitterness level	description	You think that the level of bitterness of the original liquid is about?
1	No bitterness	1	Mild	□ 1
2	Slightly bitter	2	Mild/taste slightly bitter	□ 2
3	Acceptable bitterness	3	Slight bitterness/bitterness	□ 3
4	Very bitter but still tolerable	4	Bitter/taste very bitter	□ 4
5	Unbearable bitterness	5	Extremely bitter	□ 5

Number	the effect description of taste-masking	Evaluation（"☑"）
1	Very bad. It's harder than not.	□ 1
2	Not good. With almost nothing, there is basically no concealment of bitterness.	□ 2
3	General. Better than not, bitterness has been reduced.	□ 3
4	Okay. Better than nothing, the bitterness is reduced a lot, but it is still bitter.	□ 4
5	Well. Basically not bitter!	□ 5
6	Very good. Not only not bitter, but also sweet, good!	□ 6
7	Great. I like the taste! It can be enjoyed as a drink!	□ 7

1）Bitterness: If the original liquid bitterness is 100 points, the bitterness score after adding the taste of the herbal partner is approximately:

2）Sweetness: If the sweetness of the original liquid is 0, the sweetness score after adding the taste of the herbal partner is approximately:

3）If you charge, are you willing to buy? □ Yes □ No

4）If you are willing to buy, you feel the right price is__yuan/bag.

5）other suggestion：
