# Supplementary material for: Study on Masking the Bitterness of Chinese Medicine Decoction-Mate
Source: Evid Based Complement Alternat Med. 2022 Sep 9;2022:3701288. doi: 10.1155/2022/3701288 (PMC9481366; doi:10.1155/2022/3701288)
Supplement: Supplementary Materials — Attached Table 1 is the clinical trial evaluation form, which includes the basic information of clinical subjects such as name, age, gender, and disease, as well as the description of drug bitterness, a brief introduction of filling in the form, and the options for subjects to evaluate the taste-masking effect rating of CMD-M. Attached Table 2 shows the original data of the relative retention time of the common chromatographic peaks in sample solutions before and after QRHZD taste-masking. Attached Table 3 shows the original data of the relative peak areas of the common chromatographic peaks in sample solutions before and after QRHZD taste-masking. [file 3701288.f1.zip › Attached list--Table2.pdf]

Table 2 Relative retention time of QRHZD before and after taste-masking

	S1	S2	S3	S4	S5	S6	S7	S8	S9	S10	S11	S12	S13	S14	S15	S16	S17	S18	S19	S20	RSD%
1	0.289	0.288	0.288	0.289	0.288	0.288	0.288	0.288	0.287	0.288	0.288	0.288	0.288	0.288	0.288	0.289	0.288	0.288	0.288	0.288	0.140
2	0.389	0.389	0.389	0.389	0.389	0.389	0.389	0.389	0.389	0.389	0.390	0.389	0.390	0.389	0.389	0.390	0.389	0.389	0.389	0.389	0.093
3	0.531	0.531	0.532	0.532	0.531	0.531	0.531	0.532	0.531	0.531	0.532	0.532	0.532	0.532	0.532	0.532	0.532	0.532	0.532	0.532	0.085
4	0.644	0.643	0.643	0.644	0.643	0.643	0.643	0.643	0.642	0.642	0.644	0.643	0.643	0.643	0.643	0.644	0.643	0.643	0.643	0.643	0.088
5	0.726	0.728	0.727	0.727	0.727	0.728	0.727	0.727	0.727	0.727	0.728	0.728	0.728	0.728	0.728	0.728	0.728	0.728	0.728	0.728	0.099
6	0.816	0.817	0.816	0.816	0.816	0.816	0.816	0.816	0.816	0.816	0.817	0.817	0.817	0.817	0.817	0.817	0.817	0.816	0.817	0.816	0.049
7	0.892	0.892	0.892	0.893	0.891	0.892	0.892	0.892	0.891	0.891	0.892	0.892	0.892	0.892	0.892	0.892	0.892	0.892	0.892	0.892	0.042
8	1.000	1.000	1.000	1.000	1.000	1.000	1.000	1.000	1.000	1.000	1.000	1.000	1.000	1.000	1.000	1.000	1.000	1.000	1.000	1.000	0.000
9	1.047	1.048	1.047	1.047	1.047	1.047	1.047	1.047	1.047	1.048	1.048	1.048	1.049	1.049	1.048	1.048	1.049	1.048	1.049	1.048	0.068
10	1.123	1.123	1.122	1.123	1.123	1.123	1.123	1.122	1.123	1.122	1.123	1.123	1.123	1.123	1.122	1.123	1.123	1.122	1.123	1.122	0.026
11	1.193	1.190	1.192	1.194	1.191	1.191	1.191	1.191	1.192	1.191	1.192	1.192	1.192	1.193	1.192	1.192	1.193	1.192	1.192	1.192	0.071
12	1.209	1.206	1.208	1.210	1.207	1.207	1.207	1.207	1.207	1.206	1.208	1.207	1.208	1.208	1.208	1.208	1.208	1.208	1.208	1.208	0.074
13	1.228	1.225	1.227	1.230	1.226	1.226	1.226	1.226	1.226	1.225	1.227	1.226	1.227	1.227	1.227	1.227	1.228	1.228	1.227	1.227	0.090
14	1.307	1.304	1.306	1.309	1.306	1.306	1.306	1.306	1.306	1.305	1.307	1.306	1.307	1.308	1.307	1.307	1.308	1.307	1.307	1.307	0.080
15	1.354	1.350	1.355	1.358	1.352	1.352	1.352	1.353	1.352	1.351	1.354	1.353	1.354	1.355	1.354	1.354	1.354	1.354	1.354	1.353	0.119
16	1.450	1.445	1.448	1.451	1.447	1.447	1.447	1.448	1.448	1.447	1.449	1.448	1.449	1.449	1.449	1.449	1.450	1.449	1.449	1.449	0.092
17	1.462	1.458	1.461	1.464	1.460	1.460	1.460	1.461	1.460	1.459	1.461	1.460	1.461	1.462	1.461	1.462	1.462	1.462	1.462	1.461	0.087
