# Supplementary figures and images for: Study on Masking the Bitterness of Chinese Medicine Decoction-Mate
Source: Evid Based Complement Alternat Med. 2022 Sep 9;2022:3701288. doi: 10.1155/2022/3701288 (PMC9481366; doi:10.1155/2022/3701288)

110

627

628

629

630

631

632

633

634

635

636

637

638

639

640

641 **Figures**

642 **Graphic abstract**

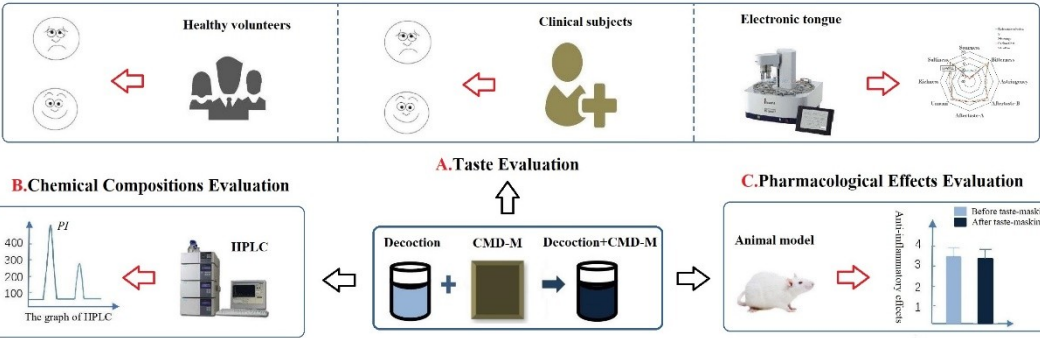

Supplement: Supplementary Materials — Attached Table 1 is the clinical trial evaluation form, which includes the basic information of clinical subjects such as name, age, gender, and disease, as well as the description of drug bitterness, a brief introduction of filling in the form, and the options for subjects to evaluate the taste-masking effect rating of CMD-M. Attached Table 2 shows the original data of the relative retention time of the common chromatographic peaks in sample solutions before and after QRHZD taste-masking. Attached Table 3 shows the original data of the relative peak areas of the common chromatographic peaks in sample solutions before and after QRHZD taste-masking. [file 3701288.f1.zip › Graphical abstract.pdf]
